# Integrating mechanical, physiological, and muscle oxygenation responses during field‐based tethered repeated‐sprint exercise in male professional soccer players

**DOI:** 10.14814/phy2.71054

**Published:** 2026-08-12

**Authors:** Fúlvia Barros Manchado‐Gobatto, Ricardo Silva Torres, Felipe Marroni Rasteiro, Allan Pinto, Pedro Paulo Menezes Scariot, Anita Brum Marostegan, João Pedro Cruz, Juan Bordon Orsi, Lara Soares de Araujo, Claudio Alexandre Gobatto

**Affiliations:** ^1^ Laboratory of Applied Sport Physiology ‐ LAFAE School of Applied Sciences, University of Campinas ‐ UNICAMP Limeira Sao Paulo Brazil; ^2^ Hypoxia, Sport and Health Team ‐ HSHT School of Applied Sciences, University of Campinas ‐ UNICAMP Limeira Sao Paulo Brazil; ^3^ Wageningen Data Competence Center Wageningen University and Research, Artificial Intelligence Group Wageningen The Netherlands; ^4^ Brazilian Synchrotron Light Laboratory ‐ LNLS, Brazilian Center for Research in Energy and Materials ‐ CNPEM Campinas Sao Paulo Brazil

**Keywords:** blood lactate, near‐infrared spectroscopy, oxygenation in less active muscle, oxygenation in locomotor muscle, repeated‐sprint exercise, running power

## Abstract

Repeated‐sprint ability is a key determinant of soccer performance. However, some mechanical and physiological responses underlying this task remain incompletely understood. This study characterized mechanical responses (force, velocity, and power) and regional muscle oxygen saturation (rSmO_2_) in the vastus lateralis (VL) and biceps brachii (BB) during field‐based tethered repeated‐sprint exercise (RSE). Associations between muscle oxygenation and mechanical performance, as well as post‐exercise rSmO_2_, heart rate (HR), and blood lactate (LAC), were also examined. Ten professional soccer players performed a tethered Running Anaerobic Sprint Test (6 × 35‐m all‐out sprints separated by 10‐s intervals), while mechanical variables and rSmO_2_ were continuously recorded. Muscle oxygenation differed according to muscle function throughout exercise and recovery. The VL exhibited greater deoxygenation during sprints, whereas the BB showed a more preserved oxygenation profile. Associations between rSmO_2_ and mechanical performance were observed only during the fifth and sixth sprints. Furthermore, only VL integrated rSmO_2_ was positively correlated with the velocity fatigue index, whereas BB oxygenation provided complementary information on the physiological adjustments associated with RSE. Post‐exercise HR and LAC were not correlated with rSmO_2_ in both muscles. Simultaneous assessment of locomotor and non‐locomotor muscles provides complementary insights into fatigue development and post‐exercise recovery RSE.

## 1 INTRODUCTION

High‐level soccer training is frequently structured around repeated‐sprint exercise (RSE) to replicate the mechanical and physiological demands encountered during match play (Chen et al., 2026). RSE consists of repeated bouts of high‐intensity sprint efforts (<10 s), interspersed with passive recovery or low‐ to moderate‐intensity activity, providing brief recovery intervals (<60 s) between sprints (Bishop et al., 2001; Girard et al., 2011; Glaister, 2005; Spencer et al., 2005). During RSE, a progressive decline in performance is commonly observed, as evidenced, for example, by reductions in power output (Akenhead et al., 2017; Sousa et al., 2015). This performance decrement is partly attributed to the disruption of oxygen (O_2_) supply homeostasis induced by repeated high‐intensity efforts (Smith & Billaut, 2010). Conversely, chronic exposure to this type of training stimulus promotes physiological adaptations that enhance tolerance to fatigue and cardiovascular and muscular stress, while improving the ability to maintain performance throughout exercise (Girard et al., 2011; Thurlow et al., 2024). Collectively, these adaptations contribute to improved performance during soccer match play (Bishop et al., 2011).

Monitoring both external and internal loads is essential for optimizing training prescription in soccer (Impellizzeri et al., 2019). To quantify external load during RSE, several approaches have been employed, including global positioning system (GPS) technology, timing systems, and devices capable of measuring the mechanical determinants of performance (Beato & Drust, 2021; Tereso et al., 2026). Among these tools, our research group developed an apparatus for tethered running condition, consisting of an instrumented sled towing capable of directly measuring the force, velocity, and power generated during sprint running (Sousa et al., 2015). This device was originally validated during RSE and is currently used to obtain direct and reliable measurements of force, velocity, power output, and fatigue index during anaerobic testing (Breda et al., 2022; Pereira et al., 2018). Furthermore, it enables continuous field‐based monitoring of force and power production throughout repeated sprints, providing a detailed assessment of the athlete's mechanical performance under sport‐specific conditions (Sousa et al., 2015). This approach overcomes the limitations of laboratory‐based assessments, which often rely on cycle ergometers or treadmills, by preserving the biomechanical specificity and natural movement patterns required in soccer, thereby enhancing the ecological validity of performance assessments conducted directly within the athletes' training environment.

Heart rate responses, blood lactate concentration, and rating of perceived exertion are among the most commonly used markers for monitoring internal training load in sport (Foster et al., 2017; Impellizzeri et al., 2019). Among these, blood lactate is a reliable indicator of exercise intensity; however, its assessment requires invasive procedure. Moreover, blood lactate kinetics are characterized by a considerable temporal delay associated with the diffusion of lactate from the contracting skeletal muscle to the bloodstream, preventing the real‐time assessment of metabolic stress (Gladden, 2004). More recently, muscle oxygenation has emerged as a promising alternative for estimating internal load in sport and exercise settings (Manchado‐Gobatto et al., 2020; Perrey et al., 2024), including in soccer (Herzog et al., 2026). Local oxygenation assessed by muscle Near‐Infrared Spectroscopy (mNIRS) enables painless, noninvasive, wearable, and high‐frequency monitoring of tissue oxygenation dynamics (Ferrari et al., 2011; Perrey et al., 2024). Unlike blood lactate measurements, which require exercise interruption and provide only discrete assessments, NIRS enables continuous, real‐time monitoring of muscle oxygenation dynamics throughout exercise, recovery intervals, and post‐exercise recovery, making it a particularly valuable tool for monitoring internal load under ecologically valid training and competition conditions.

Given the pivotal role of oxygen availability during RSE, local muscle oxygenation has become a topic of increasing interest for understanding the physiological responses to this exercise model (Archiza et al., 2020; McGawley & Bishop, 2015; Smith & Billaut, 2010). NIRS has emerged as a promising tool for investigating local muscle oxygen saturation (SmO_2_) during repeated‐sprint exercise, enabling continuous assessment throughout both sprint efforts and recovery intervals (McKee et al., 2024; Usher et al., 2025; Vasquez‐Bonilla et al., 2021; Willis et al., 2019). However, the relationship between oxygen delivery and oxygen utilization across muscles with different functional involvement in the locomotor task during RSE remains poorly understood. This knowledge gap is particularly relevant in sport‐specific settings, such as soccer, where muscles contribute differently to sprint performance and repeated high‐intensity efforts.

Among the protocols used to assess neuromuscular and metabolic performance during repeated‐sprint exercise in soccer players, the Running Anaerobic Sprint Test (RAST) is a practical field‐based protocol. The RAST consists of six maximal 35‐m sprints interspersed with 10 s of passive recovery (Zagatto et al., 2009) and provides a simple, low‐cost, and sport‐specific approach for evaluating repeated‐sprint performance. In its original form, running power is estimated exclusively from sprint time, allowing the calculation of peak, mean, and minimum power, as well as the fatigue index (Zagatto et al., 2009). Despite its widespread use, few studies have simultaneously investigated the mechanical and physiological responses elicited during the RAST. Milioni et al. (2017) reported important associations between RAST performance and the relative contribution of different metabolic pathways. However, their study was not conducted in professional soccer players, and the physiological characterization relied predominantly on invasive measurements, particularly blood lactate concentration. In contrast, Brocherie et al. (2015) demonstrated that athletes with superior repeated‐sprint performance during the RAST with active recovery exhibited greater muscle deoxygenation during the sprint bouts and faster reoxygenation during the recovery intervals. Nevertheless, in both studies, the mechanical variables were estimated indirectly, a limitation that can be overcome by the field‐based tethered running system (Sousa et al., 2015), adopted in the present study which enables the direct measurement of force, velocity, and power output during sprint running.

Finally, although the findings of Brocherie et al. (2015) significantly advanced the understanding of oxygenation dynamics in the vastus lateralis, the primary locomotor muscle involved in sprint running, an important knowledge gap remains regarding the oxygenation responses of muscles with lower functional involvement during repeated‐sprint exercise. These muscles may play an important role in local hemodynamics and physiological recovery after high‐intensity efforts (Manchado‐Gobatto et al., 2020; Osawa et al., 2017).

Considering the need to sustain high levels of running power throughout a soccer match, together with the recognized importance of oxidative metabolism during repeated‐sprint exercise, further investigation of both systemic physiological responses and local muscle oxygenation in muscles with different functional involvement during and after repeated‐ sprint exercise is warranted. Moreover, simultaneously characterizing these local and systemic physiological responses alongside direct mechanical measurements obtained with high‐frequency signal acquisition may provide novel insights into the physiological determinants of repeated‐sprint performance. Such an integrated approach has the potential to advance current knowledge in sport and exercise physiology while providing practical information for monitoring and optimizing training in soccer. Therefore, this study aimed to: (1) characterize the mechanical responses (force, velocity, and power output), regional muscle oxygen saturation (rSmO_2_) of a primary locomotor muscle (vastus lateralis, VL) and a muscle with lower functional involvement in sprint running (biceps brachii, BB), as well as heart rate responses during field‐based tethered repeated‐sprint exercise (RSE); and (2) examine the relationships between running power output and muscle oxygen saturation in both muscles throughout RSE, and between muscle oxygen saturation and post‐exercise heart rate and blood lactate concentration. We hypothesized that muscle oxygen saturation would be affected by RSE in a muscle‐specific manner, with the VL exhibiting greater deoxygenation during the sprint bouts and the BB showing greater reoxygenation during the recovery intervals. We further hypothesized that rSmO_2_ in the primary locomotor muscle would be positively associated with running power output, particularly as fatigue developed across the repeated sprints. In the post‐exercise period, we expected the VL and BB to exhibit distinct rSmO_2_ recovery patterns, reflecting their different functional involvement during RSE. We also hypothesized that muscle oxygenation would be associated with post‐exercise heart rate and blood lactate responses, providing complementary insight into local and systemic physiological recovery.

## 2 MATERIALS AND METHODS

### 2.1 Participants

Ten male professional soccer players from the second division of the state championship (age: 22 ± 1 years; body mass: 76.90 ± 7.72 kg, height: 179.70 ± 8.00 cm, and body fat: 11.07% ± 2.01%) were selected for this study.

The sample size was calculated using G*Power 3.1 software, based on a repeated‐measures ANOVA with 12 measurements per participant (interaction between time and muscle), an expected effect size of =0.30 ($\eta_{p}^{2}$ ≈ 0.08, moderate effect), *α* = 0.05, and statistical power (1 − *β*) = 0.80. The calculation indicated a minimum requirement of nine participants to detect the expected effect, corresponding to a critical *F* value of 1.90. Participants were requested to maintain their habitual dietary and hydration practices and avoid alcohol and caffeine consumption, as well as strenuous physical activity for at least 96 h prior to testing.

As an inclusion criterion, participants were required to have competed in organized soccer for at least 2 years and to be actively competing in an official state‐level championship. Players participated in structured team training six times per week in addition to official league matches, accumulating at least 20 h of training and competition‐related physical activity per week. The study was conducted in accordance with the Declaration of Helsinki and approved by the Ethics Committee of the School of Medical Sciences, University of Campinas (protocol number CAAE: 15544719.0.0000.5404). All participants provided written informed consent prior to participation in the study.

### 2.2 Study design

Three sessions were necessary to complete the study protocol. In the first and second sessions, participants were informed about the experimental design of the present study and then signed the informed consent form. Subsequently, anthropometric measurements were collected to estimate body composition. Body mass was measured using a portable digital scale (MULTILASER^®^, maximum capacity of 180 kg, precision of 100 g), while skinfold thicknesses were assessed using a skinfold caliper and circumferences were measured with a CESCORF^®^ flexible anthropometric tape measure. The Faulkner (1966) equation was used to estimate body fat percentage from the triceps, subscapular, abdominal, and suprailiac skinfold.

Following the anthropometric measurements, participants were familiarized with the tethered running apparatus used during the experimental protocol and were fitted with wearable muscle Near‐Infrared Spectroscopy (mNIRS) devices positioned over the biceps brachii (BB) and vastus lateralis (VL), as well as a heart rate monitor. The familiarization sessions were designed to minimize potential discomfort and ensure that participants became accustomed to all experimental procedures before the main testing session.

In the third session, participants performed the field‐based tethered repeated‐sprint exercise (RSE) protocol using the Running Anaerobic Sprint Test (RAST). The protocol was conducted using the tethered running apparatus described by Sousa et al. (2015), while wearing mNIRS devices for continuous monitoring of rSmO_2_ in the primary locomotor (VL) and less functionally involved (BB) muscles throughout the protocol.

**FIGURE 1:**
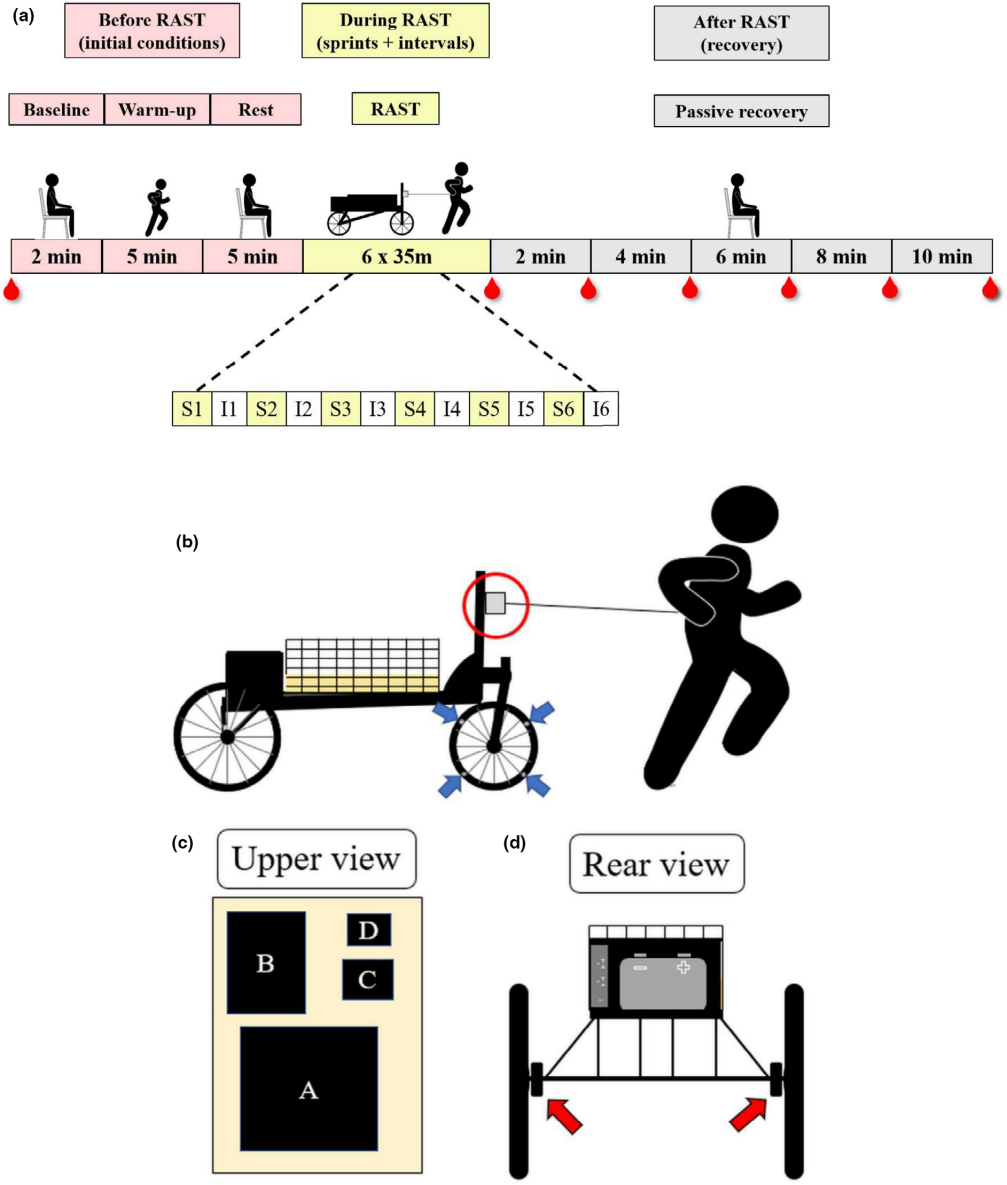
(a) The schematic overview of experimental protocol was divided in three phases, represented by the rectangles of three different colors: Yellow, indicates the before‐RAST; Red—During the RAST; and Green—After‐RAST. Heart rate (1 Hz) and muscle oxygenation (10 Hz) variables, were collected during the entire protocol. Velocity and force were measure directly (1000 Hz) throughout the RAST protocol by a tethered running apparatus. The developed running power was calculated later with the Matlab software. Samples of blood lactate concentration were collected in baseline and after‐RAST phase (i.e., every 2 min during 10 min). (b) Illustrates the participant executing a sprint tethered to the apparatus. Red circle indicates the position of the load cell; Blue arrows indicate the position of the four evenly spaced magnets. (c) Shows an upper view of the compartment that contains the components responsible for acquiring and storing the signal. A—Microcomputer; B—Power supply, responsible for supplying power/voltage into the electromagnetic brake; C—Amplifier; D—Signal acquisition module. (d) Illustrate a rear view of the tethered running apparatus, red arrows indicate the position of the electromagnetic brake.

The experimental protocol began with a 2‐min baseline recording, during which participants remained seated on a chair without armrests, maintaining the hip and knee joints at 90°. Before this recording, participants had already been resting quietly while the mNIRS devices and heart rate monitor were fitted and checked, resulting in a total resting period longer than the formal baseline recording. For subsequent analyses, the mean rSmO_2_ value recorded during the final 30s of the baseline period was used as the reference value. This was followed by a standardized warm‐up consisting of 5 min of moderate‐intensity running. Also, participants were then connected to the tethered running apparatus and performed two 35‐m familiarization sprints. Following the warm‐up procedures, participants remained seated for 5 min before performing the RAST. After completion of the test, participants remained seated for 10‐ min, during which blood samples were collected every 2 min (Figure 1a).

### 2.3 Procedures

#### 2.3.1 Tethered running anaerobic sprint test

The Running Anaerobic Sprint Test (Zagatto et al., 2009) consisted of six 35‐m sprints, interspersed by 10‐s passive recovery intervals. All tests were conducted on a natural‐grass soccer field. Participants used their own soccer shoes during all procedures. Prior to the start, participants were instructed to assume a standing sprint‐start position with the toe of one foot positioned immediately behind the starting line and to perform each sprint in an all‐out manner. After a verbal countdown provided by the evaluator, the sprint was initiated by a whistle signal. Standardized verbal encouragement was provided throughout all six sprint bouts to maintain maximal effort. Sprint time was recorded by a single investigator using a stopwatch (VL237, VOLLO, Brazil, 1/100‐s resolution) and subsequently confirmed using video recordings. All video analyses were performed by the same investigator. Lastly, for interval analyses, the 10‐s period immediately following the last sprint was considered the sixth recovery interval.

#### 2.3.2 Tethered running apparatus

The measurement of the mechanical parameters was performed using a tethered running apparatus connected to the athlete (Figure 1b) (Sousa et al., 2015). Participants wore a nylon belt commonly used for resisted sprinting and were attached to the running apparatus by an inextensible steel cable. A load cell (CSL/ZL‐250, MK CONTROL, São Paulo, Brazil) positioned on the upper front pole of the prototype and attached to the cable was used to measure the athlete's running force. This load cell was individually adjusted according to each participant's stature to maintain a horizontal orientation. As illustrated by the blue arrows in Figure 1b, four evenly spaced magnets placed in the front wheel of the tricycle were used to measure the horizontal displacement of the running apparatus. A Hall‐effect sensor fixed to the wheel axle generated one pulse each time a magnet passed the sensor (i.e., every 31 cm of horizontal displacement). Signals from the load cell and Hall‐effect sensor were acquired using an acquisition module (NI USB‐6009, NATIONAL INSTRUMENTS, Austin, USA), amplified (portable amplifier– MKTC5–10, MK CONTROL, Sao Paulo, Brazil) and stored on a microcomputer (Figure 1c). An electromagnetic brake in both rear wheels (indicated by the red arrows in Figure 1d) enabled the imposition of resistance. The resistance imposed by the tethered running apparatus was set at 19.1%. During the experimental standardization process, this level of resistance allowed the wheel to rotate without skidding while preventing the sled from reaching excessively high velocities that could compromise the tethered running effort. Signals from the load cell and Hall‐effect sensors were recorded at 1000 Hz, and processed in MATLAB environment. The load cell signal was smoothed using a fourth‐order Butterworth low‐pass filter with a cutoff frequency of 10 Hz. Conversion into Force units (N) values were obtained by linear calibration using known barbell loads attached to the load cell (acceleration of gravity considered as 9.81 m·s^−2^). As previously described, the Hall‐effect sensor provided information every 31 cm of displacement, despite the signal acquisition frequency. Displacement data were interpolated using a spline function to reach the force signal 1000‐Hz sampling frequency. The reliability and validity of the tethered running apparatus were previously demonstrated by Sousa et al. (2015).

#### 2.3.3 Muscle oxygenation measurements

Changes in muscle oxygenation were continuously assessed during baseline, exercise, and recovery periods using muscle near‐infrared spectroscopy (mNIRS). For this, two PortaMon MKII devices (ARTINIS MEDICAL SYSTEMS BV, Zetten, Netherlands) including three light source transmitters (each one with two wavelengths between 750 and 850 nm) at 30‐, 35‐, and 40‐mm distance to the receiver, were used to determine the Tissue saturation index—TSI (%), calculated by oxyhemoglobin/(oxyhemoglobin + deoxyhemoglobin) × 100, using spatially resolved spectroscopy (SRS). Throughout this manuscript, TSI is interpreted as an estimate of regional muscle oxygen saturation (rSmO_2_), in accordance with the recent terminology proposed by Feldmann et al. (2026). The NIRS devices were positioned over two muscles on the right side of the body. The biceps brachii (BB) was assessed at the midpoint of the muscle belly, with the optode positioned on the most prominent region of the muscle and centered between the proximal and distal tendon insertions to ensure optimal signal quality and consistency (Manchado‐Gobatto et al., 2020; Ogata et al., 2007; Osawa et al., 2017; Zhang et al., 2010). This muscle was selected because it has a lower functional involvement during sprint running. The vastus lateralis (VL) device was positioned 15 cm proximal to the superior border of the patella and 5 cm lateral to the midline of the thigh (Kitada et al., 2015; Manchado‐Gobatto et al., 2020; Turner et al., 2013) parallel to the long axis of the muscle (Rissanen et al., 2012). The VL was selected as the primary locomotor muscle because of its major contribution during running exercise (Guidetti et al., 1996; Woorons et al., 2019). Both mNIRS devices regions with a thin skinfold in our participants (thigh = 7.3 ± 1.8 mm, and arm (tricipital) = 6.3 ± 1.4 mm). To minimize environmental interference, the equipment was wrapped with transparent plastic to provide a waterproof barrier (Willis et al., 2019), and a dark band, to secure the probe and protect it from ambient light.

Data were sampled at 10 Hz and recorded using the manufacturer's software (OXYSOFT, ARTINIS MEDICAL SYSTEM, Netherlands). The differential path factor (DPF), a correction factor that accounts for the mean optical pathlength traveled by light within the tissue relative to the distance between the light source and detector, was adopted in the present study. Accordingly, DPF values of 3.78 for the BB and 3.83 for the VL were applied based on a previous investigation (Manchado‐Gobatto et al., 2020). After acquisition, the signals were smoothed using a 10th‐order zero‐phase low‐pass Butterworth filter (cutoff frequency = 0.1 Hz) (Woorons et al., 2019), implemented in the Artinis OXYSOFT software (Artinis Medical Systems, The Netherlands). The rSmO_2_ signals were analyzed on a second‐by‐second basis to characterize their temporal responses throughout the protocol and were also averaged across each sprint and interval for comparative analyses. The overall NIRS procedures were based on established methodological frameworks for exercise applications of the technique and adapted according to the manufacturer's recommendations and previous literature to ensure reliability and comparability of measurements.

#### 2.3.4 Physiological responses

To obtain blood lactate concentrations ([LAC]) before and after RAST (immediately after the last sprint [10‐s] and at 2, 4, 6, 8, and 10 min of recovery), blood samples (25 μL) were extracted from the earlobe (after asepsis and with the use of heparinized capillaries) and deposited in 1.5‐mL microtubes containing 400 μL of 4% trichloroacetic acid. The samples were immediately stored at 2°C–8°C [LAC] was analyzed using an enzymatic method as previously described (Engel & Jones, 1978). Absorbance was measured in a microplate reader (EPOCH, BioTek Instruments, Vermont, USA) at 340 nm against a calibration curve. To obtain individual heart rate (HR) responses before, during, and after exercise (until the 10th minute of recovery), a heart rate monitor (POLAR V800 model, Kempele, Finland) was used. HR data were recorded as RR intervals and transferred to a microcomputer via a Bluetooth interface. Heart rate data were subsequently processed and averaged for each experimental phase (baseline, exercise, and recovery) for further analysis.

### 2.4 Statistical analysis

Data were analyzed using STATISTICA version 7.0. The figures were prepared using GraphPad Prism. Data are presented as mean ± standard deviation (SD). Data normality and homogeneity of variances were initially evaluated by the Shapiro–Wilk and Levene tests, respectively.

#### 2.4.1 During tethered RSE (RAST)

A one‐way ANOVA was used to compare the mean of the mechanical variables across the six sprints (S1–S6). A two‐way repeated‐measures ANOVA for repeated measures (muscles × sprint) was performed to determine the effects of muscle (BB vs. VL) and six sprints (S1–S6) on muscle oxygen saturation. The same statistical approach was applied for the recovery intervals (muscle × interval) to determine the effects of two muscles (BB vs. VL) and six intervals (I1–I6). To quantify the overall muscle oxygenation response during tethered RSE, the area under the curve (AUC) of rSmO_2_ was calculated separately for the BB and VL by integrating the responses across all sprint bouts (S1–S6), all recovery intervals (I1–I5), and the entire RAST protocol (sprints + recovery intervals). In addition, the AUC of running power output was calculated by integrating the responses across all sprint bouts (S1–S6).

#### 2.4.2 With regard to the analysis after tethered RSE (recovery period)

A one‐way ANOVA was adopted to compare the physiological variables (blood lactate concentration [LAC] and heart rate—HR) across the different recovery time points (baseline, immediately after exercise, and 2, 4, 6, 8, and 10 min after RAST) in relation to baseline conditions. A two‐way repeated‐measures ANOVA (muscle × time) was performed to determine the effects of limb muscle (BB vs. VL) and time on muscle oxygenation responses in relation to baseline conditions.

In all analyses, the Newman–Keuls post hoc test was used to identify significant differences. Pearson's product–moment was used to determine the correlation coefficient between running power output, physiological, and muscle oxygenation variables. In all cases, the level of significance was set at *p* ≤ 0.05.

## 3 RESULTS

### 3.1 Responses throughout the experimental protocol

**FIGURE 2:**
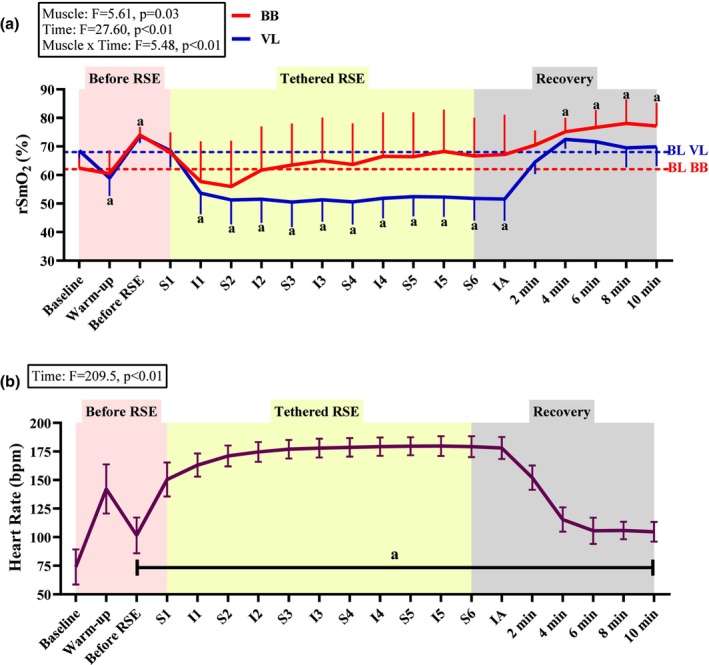
Regional muscle oxygen saturation (rSmO_2_) and heart rate responses throughout the experimental protocol. (a) Regional muscle oxygen saturation (rSmO_2_), estimated from the Tissue Saturation Index (TSI, %), in the biceps brachii (BB) and vastus lateralis (VL) over time. (b) Heart rate responses throughout the experimental protocol. Data are presented as mean ± standard deviation. a Significantly different from baseline (*p* < 0.05). *n* = 10.

With regard to the temporal responses of regional muscle oxygen saturation (rSmO_2_), a significant muscle × time interaction was observed (*p* < 0.01), along with a significant main effect of muscle (BB vs. VL= 7.90% [0.62 to 15.18%], *p* = 0.03) (Figure 2a). However, post hoc comparisons performed at each individual time point did not reveal significant differences between the BB and VL (all *p* > 0.05). Therefore, the temporal changes relative to baseline are described separately for each muscle.

In the VL (predominantly active muscle), rSmO_2_ was significantly lower than baseline during the warm‐up (Δ = −9.65%, *p* = 0.04), I1 (Δ = −14.93%, *p* < 0.01), S2 (Δ = −17.28%, *p* < 0.01), I2 (Δ = −17.03%, *p* < 0.01), S3 (Δ = −18.05%, *p* < 0.01), I3 (Δ = −17.25%, *p* < 0.01), S4 (Δ = −17.97%, *p* < 0.01), I4 (Δ = −16.71%, *p* < 0.01), S5 (Δ = −16.12%, *p* < 0.01), I5 (Δ = −16.29%, *p* < 0.01), S6 (Δ = −16.80%, *p* < 0.01), and immediately after the RAST (Δ = −17.01%, *p* < 0.01).

Conversely, the BB (less active muscle) exhibited significantly higher rSmO_2_ than baseline before the RSE (Δ = 11.63%, *p* < 0.01) and at 4 min (Δ = 12.79%, *p* < 0.01), 6 min (Δ = 14.25%, p < 0.01), 8 min (Δ = 15.72%, *p* < 0.01), and 10 min (Δ = 14.85%, *p* < 0.01) of recovery.

Regarding heart rate, one‐way repeated‐measures ANOVA revealed a significant effect of time. Post hoc analysis showed that heart rate remained significantly higher from immediately before the RSE until the 10th minute of recovery compared with baseline (Figure 2b).

### 3.2 During tethered RSE (RAST)

Mechanical variables obtained from the tethered running apparatus are presented in Table 1. Mean velocity (*r* = −0.811, *p* < 0.001), mean force (*r* = −0.533, *p* < 0.001), and mean power (*r* = −0.721, *p* < 0.001) were all significantly correlated with sprint time and progressively declined across the six sprints. Similarly, other body mass–normalized mechanical variables, including impulse and work, progressively declined, with lower values observed during the final sprints than during the initial sprints. In addition, the fatigue index (FI), calculated as [(peak − minimum)/peak × 100], was 44.4 ± 4.9%, 22.1 ± 5.5%, and 41.3 ± 13.6% for velocity, force, and power, respectively.

**TABLE 1:** Data of mechanical variables measured by tethered running apparatus measures throughout the six sprints.

	Tethered RSE (Running Anaerobic Sprint Test—RAST)						ANOVA	
	Sprint 1	Sprint 2	Sprint 3	Sprint 4	Sprint 5	Sprint 6	F	*p*
Time (s)	6.39 ± 0.45	6.87 ± 0.41^a^	7.08 ± 0.33^a^	7.58 ± 0.46^abc^	8.32 ± 0.75^abcd^	8.44 ± 0.78^abcde^	57.05	<0.001
Peak Velocity (m.s^−1^)	6.85 ± 0.32	6.61 ± 0.38	5.99 ± 0.42^ab^	5.63 ± 0.38^abc^	5.03 ± 0.38^abcd^	4.94 ± 0.31^abcd^	11.09	<0.001
Mean Velocity (m.s^−1^)	5.27 ± 0.18	5.13 ± 0.25	4.90 ± 0.28^ab^	4.49 ± 0.25^abc^	4.17 ± 0.37^abcd^	4.05 ± 0.47^abcd^	40.04	<0.001
Relative Peak Force (N.kg^−1^)	2.55 ± 0.25	2.51 ± 0.32	2.32 ± 0.28	2.31 ± 0.25	2.18 ± 0.31	2.28 ± 0.33	8.31	<0.001
Relative Mean Force (N.kg^−1^)	1.90 ± 0.19	1.72 ± 0.20^a^	1.55 ± 0.19^ab^	1.51 ± 0.17^ab^	1.42 ± 0.14^abc^	1.25 ± 0.17^abcde^	50.63	<0.001
Relative Impulse (N.kg.s^−1^)	12.13 ± 1.71	11.84 ± 1.68	11.00 ± 1.54^a^	11.44 ± 1.66	11.82 ± 1.79	10.80 ± 1.35^abe^	4.40	0.002
Relative Peak Power (N.kg^−1^)	11.84 ± 1.74	10.44 ± 1.00^a^	9.08 ± 0.91^ab^	8.39 ± 0.84^ab^	7.66 ± 1.35^abc^	7.02 ± 1.41^abcd^	8.32	<0.001
Relative Mean Power (W.kg^−1^)	9.39 ± 1.02	8.08 ± 0.84^a^	7.05 ± 0.84^ab^	6.24 ± 0.65^abc^	5.58 ± 0.88^abcd^	4.77 ± 1.10^abcde^	66.17	<0.001
Relative Work (J.kg^−1^)	59.85 ± 2.57	55.52 ± 2.06^a^	49.95 ± 2.00^ab^	47.34 ± 1.89^ab^	46.22 ± 2.20^ab^	41.10 ± 2.49^abcde^	22.89	<0.001

*Note*: Values expressed as mean ± standard deviation. Statistical analysis: a, b, c, d, e: significant differences (*p* < 0.05) in relation to 1st sprint, 2nd sprint, 3rd sprint, 4th sprint, and 5th sprint, respectively.

Abbreviations: J, Joules; J.kg^−^, Joules relativized by body mass; m.s^−1^, meters per second; N, Newtons; N.kg^−1^, Newtons relativized by body mass; W.kg^−1^, Watts relativized by body mass.

**FIGURE 3:**
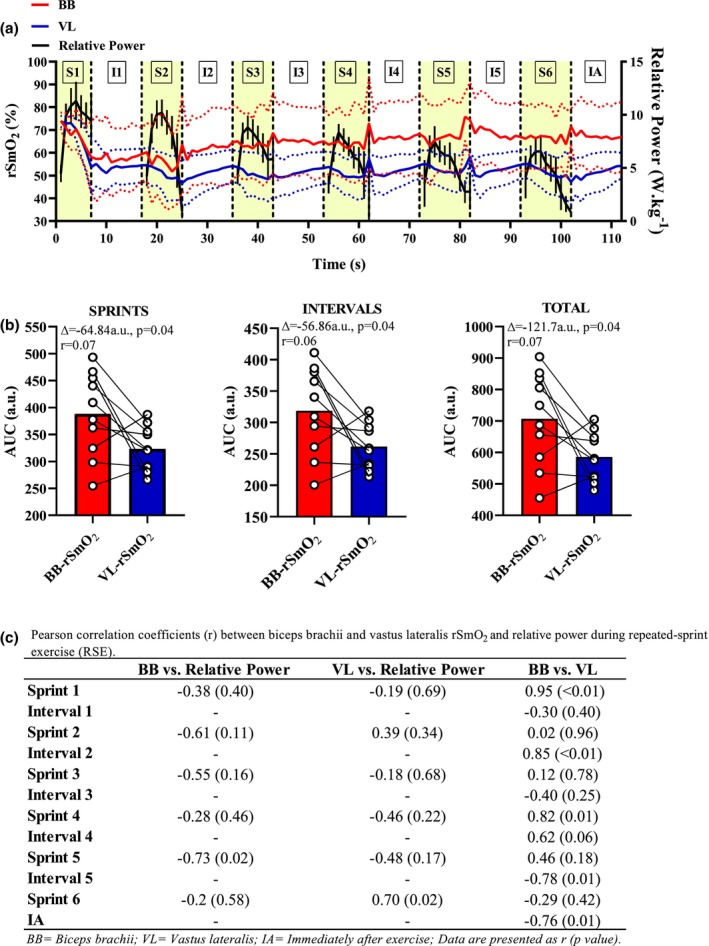
(a) Second‐by‐second mean responses of regional muscle oxygen saturation (rSmO_2_) in the biceps brachii (BB) and vastus lateralis (VL), together with relative power, during the six sprints and recovery intervals of the Running‐based Anaerobic Sprint Test (RAST). (b) Area under the curve (AUC; a.u.) of rSmO_2_ in the BB (red) and VL (blue) during the sprint phase, recovery interval phase, and the entire RAST protocol. Bars represent the mean, and dots represent individual participants. (c) Pearson correlation coefficients between relative power and rSmO_2_ in the BB and VL, calculated from the second‐by‐second mean temporal profiles for each sprint and recovery interval. *p* < 0.05; *n* = 10.

To illustrate the temporal behavior of rSmO_2_ in the BB and VL together with relative power, second‐by‐second mean values averaged across all participants (*n* = 10) were plotted throughout the RAST protocol (Figure 3a). The protocol was divided into sprint and recovery phases. Sprint duration ranged from approximately 7 to 10 s according to individual performance, whereas recovery intervals were standardized at 10 s.

Aiming to quantify the overall oxygenation response during tethered RSE, the area under the curve (AUC) of rSmO_2_ was calculated separately for both muscles by integrating the responses during all sprints (S1–S6), all recovery intervals (I1–I5), and the entire RAST protocol (sprints + recovery intervals) (Figure 3b). Significant differences between the BB and VL were observed for sprint AUC, recovery interval AUC, and total AUC (all *p* = 0.04). In all three analyses, the BB exhibited a greater AUC than the VL. However, no significant correlations were observed between the BB and VL AUC values in any of the three analyses.

To examine the correlation between muscle oxygenation and mechanical performance, Pearson's product‐moment analyses were performed. Initially, correlations between the area under the curve (AUC) of rSmO_2_ (BB and VL) and the AUC of relative power were assessed. No significant correlations were observed between the AUC of relative power and the AUC of rSmO_2_ for both muscles (*r* = 0.24 and *r* = −0.33, for BB and VL, *p* > 0.05). Subsequently, Pearson's correlations between the second‐by‐second mean profiles of relative power and rSmO_2_ (BB and VL) were calculated separately for each sprint and recovery interval using the values averaged across the 10 participants (Figure 3c). A significant negative correlation between BB rSmO_2_ and relative power was observed during Sprint 5, whereas VL rSmO_2_ was positively correlated with relative power during Sprint 6.

To further explore the relationship between mechanical fatigue and muscle oxygenation, Pearson's correlation analyses were performed between the mechanical fatigue index and the rSmO_2_ AUC values. Significant positive correlations were observed only between the FI for velocity and VL rSmO_2_ AUC during the sprint phase (*r* = 0.733, *p* < 0.05), recovery intervals (*r* = 0.729, *p* < 0.05), and the entire RAST protocol (*r* = 0.731, *p* < 0.05). No significant correlations were observed for the remaining mechanical fatigue indices or for BB rSmO_2_ AUC.

### 3.3 With regard to the analysis after tethered RSE (recovery period)

**FIGURE 4:**
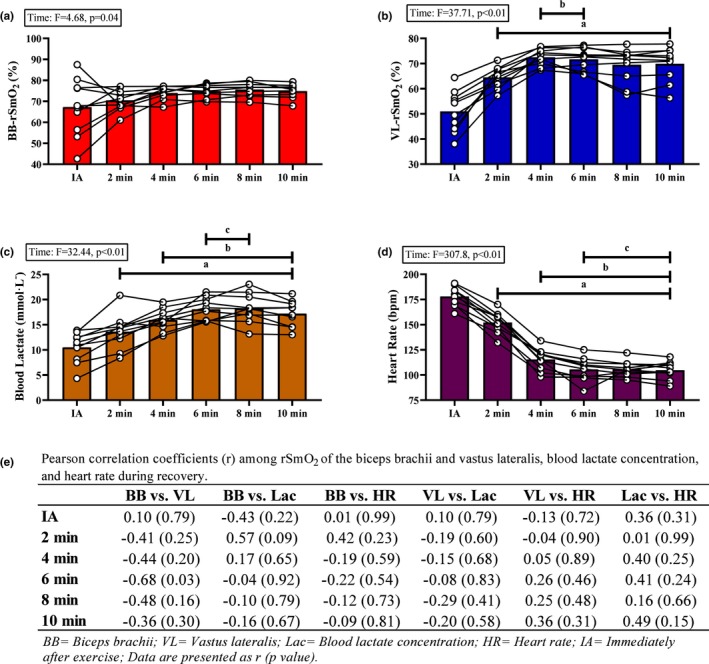
Physiological responses during recovery following the Running‐based Anaerobic Sprint Test (RAST). (a) Biceps brachii (BB) muscle oxygen saturation (rSmO_2_) over recovery time; (b) Vastus lateralis (VL) muscle oxygen saturation (rSmO_2_) over recovery time; (c) Blood lactate concentration over recovery time; (d) Heart rate over recovery time; (e) Pearson correlation coefficients (*r*) among the physiological variables. Data are presented as mean with individual values (*n* = 10). a = significantly different from baseline; b = significantly different from 2 min; c = significantly different from 4 min. *p* < 0.05.

The variables assessed during the 10‐min recovery period following the RSE included rSmO_2_, estimated from the TSI, in the BB (Figure 4a) and VL (Figure 4b), blood lactate concentration (Figure 4c), and heart rate (Figure 4d). A significant effect of time was observed for all variables (all *p* < 0.05). Specifically, VL rSmO_2_ differed between the immediate post‐RSE measurement and all subsequent recovery time points (2–10 min) (all *p* < 0.01), as well as between 2 and 4 min and between 2 and 6 min (both *p* < 0.01). Similarly, LAC differed between the immediate post‐RSE measurement and all subsequent recovery time points (2–10 min) (all *p* < 0.05), between 2 min and 4–10 min (all *p* < 0.01), and between 4 min and 6–8 min (both *p* < 0.05). Peak blood lactate concentration reached 18.78 ± 2.41 mM. Heart rate also differed between the immediate post‐RSE measurement and all subsequent recovery time points (2–10 min) (all *p* < 0.01), between 2 min and 4 and 10 min (both *p* < 0.01), and between 4 min and 6–10 min (all *p* < 0.05). At the 10th min of recovery, the mean changes relative to baseline were +15.88 mM for blood lactate concentration, +31 bpm for heart rate, +12.71% for BB rSmO_2_, and +1.34% for VL rSmO_2_. No significant correlations were observed among the Δ values of the recovery variables. Subsequently, Pearson's correlation analyses were performed separately at each recovery time point (immediately after, 2, 4, 6, 8, and 10 min). A significant inverse correlation was observed only between BB and VL rSmO_2_ at the 6‐min recovery time point (Figure 4e).

## 4 DISCUSSION

First, this study sought to advance current knowledge regarding the integration of mechanical, systemic physiological, and local physiological (muscle oxygenation) responses during and following repeated high‐intensity exercise. To the best of our knowledge, this is the first study to simultaneously and directly quantify force, velocity, and power together with regional muscle oxygen saturation (rSmO_2_), estimated from the Tissue Saturation Index (TSI), in muscles with different levels of involvement in running, before, during, and after tethered repeated‐sprint exercise, specifically the Running Anaerobic Sprint Test. Furthermore, this is the first investigation to examine the correlations between directly measured mechanical parameters, local muscle oxygenation responses, and systemic physiological variables throughout exercise and recovery in soccer players. The main findings demonstrated that rSmO_2_ exhibited a muscle‐specific pattern during the sprint bouts, recovery intervals, and post‐exercise recovery. While the vastus lateralis displayed greater deoxygenation during exercise and a toward stabilization of rSmO_2_ during the final sprints, the biceps brachii exhibited a more preserved oxygenation profile and greater integrated reoxygenation (AUC) throughout the protocol. Furthermore, only the integrated rSmO_2_ response in the vastus lateralis (VL) muscle—during sprints, interval, and the test as a whole—showed a significant, positive association with the velocity fatigue index, indicating the relevance of deoxygenation at this site for maintaining running velocity. This correlation provides complementary information regarding physiological responses to high‐intensity intermittent exercise.

Our experimental design enabled continuous monitoring of rSmO_2_, estimated from TSI, in two muscles with distinct functional roles during running, as well as heart rate, from baseline through 10 min of recovery following the RSE (Figure 2). These variables were selected because they allow continuous monitoring, using wearable devices, of both local oxygen delivery and utilization dynamics within skeletal muscle and the systemic cardiovascular response to exercise. Although HR has limited sensitivity for distinguishing physiological changes between successive sprint bouts, it remains one of the most widely used indicators of internal training load in soccer (Tereso et al., 2026). Furthermore, all measurements were obtained under ecologically valid conditions during a field‐based testing session, thereby preserving the specific characteristics of the sport.

In the present study, rSmO_2_ was estimated from the TSI obtained using two PortaMon MK II devices (Artinis Medical Systems, The Netherlands). Among the variables provided by wearable near‐infrared spectroscopy (mNIRS), TSI has been considered one of the most robust indicators of muscle oxygenation status in experimental settings where blood flow changes dynamically, as occurs during repeated high‐intensity exercise (Brocherie et al., 2015; Wolf et al., 2007). Accordingly, considering our experimental design and recent methodological recommendations (Feldmann et al., 2026), TSI was interpreted as an estimate of rSmO_2_. Moreover, when current methodological recommendations are carefully followed (Feldmann et al., 2026; Perrey et al., 2024), wearable mNIRS devices represent a practical, noninvasive, and ecologically valid tool for continuously monitoring local oxygen delivery and utilization dynamics in skeletal muscle. In this context, the simultaneous assessment of muscles with different levels of functional involvement, as proposed in the present study and previously explored by other investigators (Cirino et al., 2025; Rissanen et al., 2012; Sendra‐Pérez et al., 2025; Yogev et al., 2022, 2023), broadens the understanding of the integration between local and systemic physiological responses during high‐intensity exercise and represents a promising approach for future research and practical applications in sports monitoring.

### 4.1 Muscle oxygenation during tethered RSE

As shown in Figure 2a, both muscles exhibited similar rSmO_2_ values (~73%) before the RAST, indicating that both muscles started the exercise under comparable oxygenation conditions. Furthermore, continuous monitoring captured the small fluctuations in muscle oxygenation that occurred during the warm‐up and the post‐warm‐up period, confirming the stability of both muscles before the onset of exercise. Therefore, the differences observed throughout the protocol are likely attributable to exercise‐induced physiological adjustments rather than pre‐existing differences in muscle oxygenation status.

During the RAST, however, a clear muscle‐specific pattern emerged. The vastus lateralis exhibited a rapid decline in rSmO_2_ during the initial sprint bouts, followed by only partial reoxygenation during the 10‐s passive recovery intervals and a subsequent stabilization of muscle oxygenation during the final sprints. This pattern suggests a progressive mismatch between the high metabolic demand imposed on the primary locomotor muscle and the limited time available for restoring oxygen availability between successive efforts. Consequently, as the sprint bouts accumulated, the recovery intervals became insufficient to fully re‐establish the balance between oxygen delivery, extraction, and utilization before the onset of the subsequent sprint. The stabilization of rSmO_2_ observed during the final repetitions may further indicate that the active locomotor muscle approached a physiological ceiling for peripheral oxygen extraction, whereby additional reductions in muscle oxygenation became minimal despite the continued decline in mechanical performance. These findings are consistent with those reported by Brocherie et al. (2015), who also observed a rapid decrease in vastus lateralis oxygenation accompanied by incomplete reoxygenation during a RAST protocol performed by international male soccer players. Although methodological differences exist between the two studies—particularly regarding the placement of the mNIRS sensor on the vastus lateralis and the use of tethered running in the present investigation—the overall physiological response was remarkably similar. Together, these findings reinforce that incomplete muscle reoxygenation is a characteristic feature of repeated high‐intensity exercise performed with short recovery intervals.

Comparable responses were also reported by Vasquez‐Bonilla et al. (2021), who evaluated female soccer players performing a repeated‐sprint protocol (8 × 20 m, 20‐s recovery intervals) while monitoring gastrocnemius rSmO_2_ using wearable mNIRS. Despite differences in participant sex, the muscle evaluated, sprint distance, and recovery duration, the authors likewise reported progressive muscle deoxygenation during the sprint bouts accompanied by only partial reoxygenation between repetitions. Based on these observations, they highlighted the need for future studies integrating mNIRS‐derived variables with indicators of mechanical workload, vascular hemodynamics, and metabolic energy pathways. The present study extends this perspective by simultaneously combining muscle oxygenation measurements in muscles with distinct functional roles, directly measured mechanical parameters acquired at a high sampling frequency, and continuous physiological monitoring under ecologically valid field conditions while maintaining rigorous experimental control.

Taken together, these findings indicate that the locomotor muscle primarily reflects the progressive increase in local metabolic demand throughout repeated‐sprint exercise. Whether muscles with lower mechanical involvement provide complementary physiological information beyond that obtained from the locomotor musculature is addressed in the following section.

One of the most interesting findings of the present study was the distinct muscle oxygenation behavior observed between the locomotor muscle (vastus lateralis, VL) and the non‐locomotor muscle (biceps brachii, BB) throughout the RAST. This difference was consistently observed both in the mean rSmO_2_ responses recorded during each sprint and recovery interval (Figure 2), in the temporal profiles, and area under the curve (AUC) analyses (Figure 3a,b). Whereas the VL exhibited marked deoxygenation throughout the repeated‐sprint bouts, the BB maintained mean rSmO_2_ values close to those observed at baseline.

Moreover, the BB displayed a distinct oxygenation pattern during the brief recovery intervals. In contrast to the VL, rSmO_2_ progressively increased in the BB throughout the recovery periods between sprint bouts. This response suggests that the transient reduction in metabolic demand during the intervals promotes systemic physiological adjustments that extend beyond the recovery of the primary locomotor muscle, also involving muscles with lower mechanical involvement in the task. This interpretation is supported by the findings of Bae et al. (2000), who demonstrated simultaneous increases in pulmonary oxygen uptake (VO_2_) and VL rSmO_2_ during the recovery intervals of intermittent high‐intensity exercise. Although part of this increase reflects enhanced oxygen uptake by the active musculature, the authors proposed that the elevated VO_2_ also represents repayment of the oxygen deficit, involving additional oxygen consumption by less active muscles and other tissues not directly engaged in the exercise (Camus & Thys, 1991; Di Prampero, 1981). Within this context, our findings suggest that the BB response may, at least in part, reflect these systemic physiological adjustments, including the transient redistribution of blood flow and the restoration of the balance between oxygen delivery and oxygen utilization during the recovery intervals. Although the wearable mNIRS devices used in the present study provide estimates of total hemoglobin (tHb), a variable incorporated into the calculation of TSI, future investigations should combine these measurements with more direct assessments of blood flow, such as Doppler ultrasonography. Such an approach may provide a more comprehensive characterization of the hemodynamic mechanisms underlying the distinct oxygenation responses observed in muscles with different functional roles during repeated high‐intensity exercise.

Collectively, these findings indicate that monitoring muscles with distinct functional roles provides complementary physiological information regarding the interaction between local metabolic demand and systemic recovery during repeated‐sprint exercise.

### 4.2 Mechanical responses and integration with muscle oxygenation

Beyond the simultaneous assessment of muscle oxygenation in two muscles with distinct functional roles, the present study also advances previous soccer‐related investigations by providing direct field‐based measurements of force, velocity, and running power during tethered repeated‐sprint exercise. The instrumented tethered running system additionally enabled the calculation of force‐, velocity‐, and power‐specific fatigue indices, variables that are rarely obtainable under ecologically valid field conditions. From a practical perspective, these measurements allow practitioners to determine whether the progressive decline in running power is predominantly associated with impairments in force production or movement velocity, thereby supporting more individualized training interventions.

As expected during repeated all‐out efforts, mechanical performance progressively declined throughout the protocol. Running power increased rapidly at the beginning of each sprint but could not be sustained over the 35‐m distance. Sprint time increased after the third repetition, whereas peak power was generally achieved during the first or second sprint and reached its lowest values in the sixth sprint. Similar findings have been reported using the same tethered running system in trained sprinters (Sousa et al., 2015), although differences were observed in peak velocity, power, and relative work. These differences may be partly explained by the athletes' characteristics (sprinters vs. soccer players), the testing surface (athletics track vs. natural‐grass soccer field), and the resistance applied to the tethered running system, which was adjusted to ensure reliable mechanical measurements under the present experimental conditions. Collectively, these findings support the validity of the methodology, demonstrate its applicability to sport‐specific testing environments, and confirm the sensitivity of these mechanical variables to distinguish athletes according to the physiological and mechanical demands of their sport.

A distinctive feature of the present investigation was the simultaneous visualization of mechanical power output together with muscle oxygenation responses throughout both the sprint and recovery phases (Figure 3a). This integrated representation provides a dynamic overview of the interaction between external load (mechanical power output) and internal physiological responses (muscle oxygenation), allowing changes in performance and local oxygen availability to be interpreted simultaneously throughout the entire repeated‐sprint protocol. Such an approach may prove valuable for monitoring individual adaptations to training interventions and for identifying subtle physiological changes that are not evident when mechanical or physiological variables are analyzed separately.

To further investigate the relationship between mechanical performance and muscle oxygenation, we analyzed the area under the curve of both physiological and mechanical responses, as well as their respective correlations. Although the BB consistently exhibited higher rSmO_2_ AUC values than the VL during both sprint and recovery periods, no significant correlation was observed between the two muscles when considering the entire protocol. Likewise, no significant relationship was identified between overall running power and muscle oxygenation when the complete RSE was analyzed. Rather than indicating an absence of physiological interaction, these findings likely reflect the substantial interindividual variability in muscle oxygenation responses, highlighting the sensitivity of wearable mNIRS devices to detect individual physiological profiles and reinforcing their potential application for tailored monitoring of training adaptations.

Interestingly, significant point‐by‐point correlations between muscle oxygenation and mechanical performance emerged only during the final bouts of the protocol (Figure 3c). During the sixth sprint, a positive correlation was observed between VL oxygenation and relative running power, suggesting that local oxygen availability becomes progressively more relevant for sustaining mechanical performance as neuromuscular fatigue develops. Likewise, the positive association between the AUC of VL rSmO_2_ and the velocity fatigue index indicates that athletes exhibiting a distinct oxygenation profile in the primary locomotor muscle also experienced greater reductions in sprinting velocity across repeated efforts. Although this relationship does not imply causality, it highlights the close interaction between local oxygenation dynamics and the mechanical manifestations of fatigue during repeated‐sprint exercise. During the initial sprints, when phosphocreatine availability is relatively preserved and mechanical output remains high, muscle oxygenation is likely to play a less limiting role. However, as repeated sprints progressively challenge metabolic homeostasis and phosphocreatine resynthesis, local oxygen availability may become increasingly important for sustaining mechanical performance. This interpretation extends previous observations demonstrating associations between muscle oxygenation dynamics and repeated‐sprint performance (Billaut & Buchheit, 2013; Smith & Billaut, 2010).

On the other hand, an inverse correlation was identified between BB oxygenation and relative running power during the fifth sprint, indicating that athletes with greater BB reoxygenation tended to exhibit lower mechanical performance. This heterogeneous behavior between muscles with distinct functional roles further reinforces the concept that the BB provides complementary physiological information regarding the local and systemic hemodynamic adjustments occurring during repeated‐sprint exercise. In a previous study involving a 30‐s all‐out continuous run (AO30), distinct oxygenation responses were likewise observed between the more active and less active muscles (Manchado‐Gobatto et al., 2020). However, unlike the responses observed during the present RSE protocol, lower TSI values were recorded in the BB than in the VL during the AO30, whereas the arm muscle exhibited a faster reoxygenation response following exercise (Manchado‐Gobatto et al., 2020). Taken together, these findings suggest that the oxygenation responses of less active muscles are strongly influenced by the characteristics of the exercise task and highlight the importance of simultaneously assessing muscles with different functional roles to obtain a more comprehensive understanding of the physiological mechanisms underlying performance during high‐intensity exercise.

From a practical perspective, the present findings suggest that wearable muscle oxygenation devices may complement traditional mechanical assessments by identifying individualized physiological responses associated with fatigue development during repeated‐sprint exercise. Such information may contribute to more personalized training prescription, optimization of recovery strategies, and potentially earlier identification of athletes presenting altered physiological responses that could increase susceptibility to excessive fatigue or musculoskeletal injury.

Finally, an additional methodological advantage of the present study lies in the use of an instrumented tethered running system. Unlike the conventional Running Anaerobic Sprint Test, in which running power is estimated from body mass and sprint time (Zagatto et al., 2009), the tethered approach provides direct measurements of horizontal force, running velocity, and mechanical power throughout each sprint. Furthermore, continuous second‐by‐second recording substantially expands the analytical potential of repeated‐sprint testing, allowing a more detailed characterization of fatigue development and enabling integrated analyses between mechanical performance and physiological responses. Collectively, these methodological advances considerably strengthen the assessment of repeated‐sprint ability and provide a comprehensive framework for future investigations examining fatigue mechanisms and training adaptations in team‐sport athletes.

### 4.3 Recovery following tethered repeated‐sprint exercise

Regarding the responses following RSE, our hypotheses were partially confirmed, as oxygenation in the two muscles did not exhibit the same behavior. However, we did not observe significant correlations between rSmO_2_ in the BB and VL with heart rate and lactate concentration. After the tethered, both muscles exhibited progressive reoxygenation during the recovery period, although with distinct temporal patterns. The VL recovered rapidly, reaching a plateau approximately 2 min after exercise, maintaining rSmO_2_ values similar to baseline throughout the remainder of the monitored recovery period. In contrast, the BB displayed a slower and more gradual reoxygenation profile, reaching a plateau only around the eighth minute of recovery. Notably, only the BB exhibited rSmO_2_ values that significantly exceeded baseline, beginning approximately 4 min after exercise and remaining elevated until the end of the 10‐min recovery period. These findings further reinforce the concept that muscles with distinct functional roles exhibit complementary physiological responses not only during repeated‐sprint exercise but also throughout post‐exercise recovery.

The distinct recovery kinetics observed between the VL and BB likely reflect differences in the physiological mechanisms regulating oxygen delivery and utilization following RSE, an exhaustive exercise. Whereas the locomotor muscle rapidly restores oxygen availability to support phosphocreatine resynthesis and metabolic recovery, the prolonged reoxygenation observed in the BB may reflect regional redistribution of blood flow toward tissues with lower metabolic demand as systemic homeostasis is progressively restored. This interpretation is consistent with previous evidence demonstrating that recovery from intense exercise involves complex cardiovascular adjustments, including autonomic regulation, changes in vasomotor tone, and regional redistribution of blood flow (Halliwill et al., 2013; Joyner & Casey, 2015).

Our findings are also consistent with previous investigations using wearable NIRS technology. Arnold et al. (2024) reported a similar pattern of muscle reoxygenation during incremental cycling exercise, whereas Jones et al. (2013) demonstrated considerable interindividual variability in gastrocnemius rSmO_2_ recovery following repeated‐sprint exercise. In that study, some individuals exhibited rapid restoration of muscle oxygenation, whereas others failed to recover baseline values even after 3 min of recovery. Together with the present results, these observations reinforce the sensitivity of wearable mNIRS devices to detect individualized physiological recovery profiles, supporting their application for personalized monitoring of training adaptations in sport settings.

Interestingly, although the overall recovery profile followed the expected physiological pattern, an inverse correlation between VL and BB rSmO_2_ was observed only during the sixth minute of recovery. This finding further supports the concept that muscles with distinct functional roles recover asynchronously, likely reflecting differences in local metabolic demand and systemic hemodynamic regulation rather than a uniform restoration of oxygen homeostasis throughout the body.

The recovery period was also characterized by the expected systemic physiological responses. Heart rate progressively declined, with significant reductions observed until approximately the sixth minute of recovery, whereas blood lactate concentration continued to increase, reaching peak values between 6 and 8 min after exercise, depending on the individual. The peak blood lactate concentration (18.78 ± 2.41 mmol·L^−1^) confirms the high metabolic demand imposed by the repeated‐sprint protocol. Beyond being a classical marker of anaerobic glycolytic contribution, lactate should also be interpreted as an important intermediary metabolite linking glycolytic ATP production with mitochondrial oxidative metabolism (Brooks, 2018). Therefore, the temporal dissociation between heart rate recovery, muscle reoxygenation, and peak blood lactate further illustrates the complexity of the physiological processes involved in restoring homeostasis following repeated‐sprint exercise.

Finally, within our experimental design, no significant correlations were identified between muscle oxygenation and traditional internal load markers commonly used in soccer—such as heart rate and blood lactate concentration. This suggests that wearable mNIRS technology provides physiological information complementary to conventional monitoring tools by simultaneously capturing local muscle oxygenation. This lack of association may stem from the sensitivity of mNIRS in detecting more subtle individual variations regarding recovery from high‐intensity intermittent exercise stimuli. Thus, for future applications, we suggest that an integrated approach combining local oxygenation measurements with the analysis of systemic physiological responses could contribute to a more individualized assessment of recovery status, the optimization of training prescription, and the identification of athletes exhibiting atypical physiological responses following high‐intensity intermittent exercise. Taken together, these results demonstrate that integrating continuous mechanical measurements with the simultaneous monitoring of oxygenation in muscles with distinct functional roles offers a comprehensive framework for understanding fatigue development and post‐exercise recovery during repeated‐sprint activities.

## 5 FINAL CONSIDERATIONS

The present study provides novel insights into the mechanical and physiological responses to tethered repeated‐sprint exercise in soccer players by integrating continuous direct measurements of force, velocity, and running power with simultaneous monitoring of oxygenation in muscles with distinct functional roles. The findings demonstrate that the locomotor muscle (vastus lateralis) and the non‐locomotor muscle (biceps brachii) exhibit distinct oxygenation dynamics during both exercise and recovery, indicating complementary physiological responses associated with local metabolic demand and systemic hemodynamic adjustments.

Furthermore, the integration of continuous mechanical measurements with wearable muscle near‐infrared spectroscopy revealed that local muscle oxygenation became increasingly associated with mechanical performance as fatigue developed throughout repeated‐sprint exercise. During recovery, the distinct reoxygenation kinetics observed between muscles further highlighted the complexity of the physiological mechanisms involved in restoring homeostasis following maximal intermittent exercise. Overall, the present study expands current understanding of the interaction between mechanical performance and muscle oxygenation during repeated‐sprint exercise and highlights the value of assessing muscles with distinct functional roles to obtain complementary physiological information regarding fatigue development and post‐exercise recovery.

### 5.1 Limitations

The present study provides novel insights into the mechanical and physiological responses to tethered repeated‐sprint exercise. However, several limitations should be acknowledged. First, skinfold thickness was not measured at the exact anatomical site where the wearable mNIRS device was positioned over the biceps brachii. Nevertheless, this limitation is unlikely to have materially influenced the interpretation of the regional muscle oxygen saturation data, as all participants were eutrophic soccer players with low body fat percentages and low skinfold thickness, particularly in the upper body. For example, the triceps skinfold thickness (6.3 ± 1.4 mm) was well below the threshold considered likely to affect the accuracy of the mNIRS device used in the present study. According to McCully and Hamaoka (2000), subcutaneous adipose tissue thickness should be less than approximately half the source–detector separation distance. Considering the maximum source–detector separation of 40 mm used in the present study, this corresponds to approximately 20 mm. Furthermore, mNIRS signal quality was continuously monitored using the fit factor provided by the Artinis system. This parameter ranges from 0 to 100, with higher values indicating a better fit of the acquired optical signal. In the present study, all measurements were obtained with fit factor values consistently above 99.0, indicating excellent signal quality and further supporting the reliability of the recorded TSI data.

Although wearable mNIRS provides valuable information regarding local muscle oxygenation, the simultaneous assessment of pulmonary oxygen uptake (V̇O_2_) would further improve the interpretation of the interaction between systemic oxygen delivery and local muscle oxygen utilization during both exercise and recovery. Future studies should therefore combine pulmonary gas exchange measurements with wearable mNIRS‐derived muscle oxygenation, together with cerebral oxygenation monitoring, to provide a more integrated understanding of oxygen transport and utilization during RSE (Orcioli‐Silva et al., 2024; Simpson et al., 2025).

Furthermore, the present findings indicate that the recovery period following RSE contains important physiological information that deserves further investigation, particularly when the Running Anaerobic Sprint Test (RAST) is applied to soccer players (Michailidis et al., 2020). Future studies may also benefit from incorporating Doppler ultrasonography to characterize post‐exercise hemodynamic responses more comprehensively and improve the understanding of vascular regulation during recovery. Such multimodal approaches may further clarify the physiological mechanisms underlying fatigue development and post‐exercise recovery during repeated‐sprint exercise.

### 5.2 Practical applications

The present findings demonstrate that continuous monitoring of muscle oxygen saturation using wearable near‐infrared spectroscopy provides valuable physiological information that complements traditional internal load markers, such as heart rate and blood lactate concentration, during repeated‐sprint exercise performed under ecologically valid field conditions. Moreover, the distinct oxygenation patterns observed between the primary locomotor muscle (vastus lateralis) and the non‐locomotor muscle (biceps brachii) indicate that muscles with different functional roles exhibit complementary physiological responses during both exercise and recovery.

Furthermore, the marked interindividual variability observed in muscle oxygenation responses reinforces the sensitivity of wearable mNIRS for identifying individualized physiological profiles. These findings indicate that athletes may exhibit distinct physiological recovery patterns despite performing the same repeated‐sprint protocol, highlighting the importance of individualized monitoring rather than relying exclusively on group‐average responses. Considering that muscle oxygenation was also associated with repeated‐sprint performance, wearable mNIRS may represent a valuable tool for monitoring internal training load, evaluating recovery status, and supporting individualized training prescription in soccer players.

The present findings further suggest that monitoring physiological responses during recovery intervals may provide information that is as valuable as monitoring exercise performance itself. The observation that meaningful physiological recovery occurred even during 10‐s recovery intervals highlights the importance of individual recovery dynamics and may assist coaches and practitioners in optimizing repeated‐sprint training, recovery strategies, and training load management.

Overall, integrating wearable muscle oxygenation monitoring with continuous direct mechanical assessment and traditional physiological markers provides a robust and multidimensional framework for evaluating fatigue development, recovery dynamics, and repeated‐sprint performance under field conditions. This integrated approach has the potential to improve individualized training prescription, optimize recovery strategies, and enhance performance monitoring in soccer and other intermittent team sports.

## AUTHOR CONTRIBUTIONS

**Fúlvia B. Manchado‐Gobatto:** Conceptualization; data curation; formal analysis; funding acquisition; investigation; methodology; project administration; supervision. **Ricardo Silva Torres:** Funding acquisition; investigation; supervision. **Felipe Marroni Rasteiro:** Conceptualization; formal analysis; investigation; methodology. **Allan Pinto:** Formal analysis; methodology; software. **Pedro P. Menezes Scariot:** Investigation; methodology. **Anita Brum Marostegan:** Formal analysis; methodology. **João Pedro Cruz:** Formal analysis; methodology. **Juan Bordon Orsi:** Formal analysis; investigation; methodology. **Lara Soares Araujo:** Data curation; formal analysis; investigation. **Claudio A. Gobatto:** Conceptualization; data curation; formal analysis; funding acquisition; investigation; methodology; project administration; supervision.

## FUNDING INFORMATION

The study was partially supported by São Paulo Research Foundation—FAPESP (2016/50250‐1, 2018/05821‐6, 2019/02286‐5, 2019/20894‐2, 2019/10666‐2, 2019/16253‐1, 2023/02728‐3, 2024/04688‐1, 2024/03587‐7), the National Council for Scientific and Technological Development—CNPq (308117/2018‐2, 309832/2021‐7, 409521/2021‐3, 444434/2024‐0), FAEPEX – UNICAMP (2583/20), and Coordenação de Aperfeiçoamento de Pessoal de Nível Superior—Brasil (CAPES)—Finance Code 001.

## CONFLICT OF INTEREST STATEMENT

The authors below declare to have no conflicts of interest to declare that are relevant to the content of this article. The authors also certify that they have no affiliations with or involvement in any organization or entity with any financial interest or non‐financial interest in the subject matter or materials discussed in this manuscript.

## ETHICS STATEMENT

The study was conducted in accordance with the Declaration of Helsinki and approved by the Ethics Committee of The School of Medical Sciences, located at the University of Campinas (protocol number – CAAE—15544719.0.0000.5404).

## Data Availability

The source data for this study are available upon reasonable request at https://drive.google.com/drive/folders/1I62WjP9GtkvPGr0M_Rco8LqqEj2iiNrD?usp=sharing.

## References

[phy271054-bib-0002] Akenhead, R. , Marques, J. B. , & Paul, D. J. (2017). Accelerometer load: A new way to measure fatigue during repeated sprint training? Science and Medicine in Football, 1(2), 151–156. 10.1080/24733938.2017.1330550

[phy271054-bib-0003] Archiza, B. , Andaku, D. K. , Beltrame, T. , Libardi, C. A. , & Borghi‐Silva, A. (2020). The relationship between repeated‐Sprint ability, aerobic capacity, and oxygen uptake recovery kinetics in female soccer athletes. Journal of Human Kinetics, 75(1), 115–126. 10.2478/hukin-2020-0042 33312300 PMC7706679

[phy271054-bib-0004] Arnold, J. I. , Yogev, A. , Nelson, H. , van Hooff, M. , & Koehle, M. S. (2024). Muscle reoxygenation is slower after higher cycling intensity, and is faster and more reliable in locomotor than in accessory muscle sites. Frontiers in Physiology, 15, 1449384. 10.3389/fphys.2024.1449384 39206382 PMC11349675

[phy271054-bib-0005] Bae, S. Y. , Hamaoka, T. , Katsumura, T. , Shiga, T. , Ohno, H. , & Haga, S. (2000). Comparison of muscle oxygen consumption measured by near infrared continuous wave spectroscopy during supramaximal and intermittent pedalling exercise. International Journal of Sports Medicine, 21(3), 168–174. 10.1055/s-2000-8880 10834347

[phy271054-bib-0006] Beato, M. , & Drust, B. (2021). Acceleration intensity is an important contributor to the external and internal training load demands of repeated sprint exercises in soccer players. Research in Sports Medicine (Print), 29(1), 67–76. 10.1080/15438627.2020.1743993 32200649

[phy271054-bib-0007] Billaut, F. , & Buchheit, M. (2013). Repeated‐sprint performance and vastus lateralis oxygenation: Effect of limited O_2_ availability. Scandinavian Journal of Medicine & Science in Sports, 23(3), e185–e193. 10.1111/sms.12052 23362832

[phy271054-bib-0008] Bishop, D. , Girard, O. , & Mendez‐Villanueva, A. (2011). Repeated‐sprint ability ‐ part II: Recommendations for training. Sports Medicine (Auckland, N.Z.), 41(9), 741–756. 10.2165/11590560-000000000-00000 21846163

[phy271054-bib-0009] Bishop, D. , Spencer, M. , Duffield, R. , & Lawrence, S. (2001). The validity of a repeated sprint ability test. Journal of Science and Medicine in Sport, 4(1), 19–29. 10.1016/S1440-2440(01)80004-9 11339490

[phy271054-bib-0010] Breda, F. , Breda, F. L. , Manchado‐Gobatto, F. B. , Barros Sousa, F. A. , Beck, W. R. , Pinto, A. , Papoti, M. , Scariot, P. P. M. , & Gobatto, C. A. (2022). Complex networks analysis reinforces centrality hematological role on aerobic‐anaerobic performances of the Brazilian Paralympic endurance team after altitude training. Scientific Reports, 12(1), 1148. 10.1038/s41598-022-04823-w 35064131 PMC8782909

[phy271054-bib-0011] Brocherie, F. , Millet, G. P. , & Girard, O. (2015). Neuro‐mechanical and metabolic adjustments to the repeated anaerobic sprint test in professional football players. European Journal of Applied Physiology, 115(5), 891–903. 10.1007/s00421-014-3070-z 25481506

[phy271054-bib-0012] Brooks, G. A. (2018). The science and translation of lactate shuttle theory. Cell Metabolism, 27(4), 757–785. 10.1016/j.cmet.2018.03.008 29617642

[phy271054-bib-0014] Camus, G. , & Thys, H. (1991). An evaluation of the maximal anaerobic capacity in man. International Journal of Sports Medicine, 12(4), 349–355. 10.1055/s-2007-1024693 1917217

[phy271054-bib-0015] Chen, A. , Zhuang, M. , Huang, Z. , Zhao, L. , & Wang, L. (2026). The effects of field‐based repeated sprint training on physical performance in soccer players: A systematic review and multilevel meta‐analysis. Frontiers in Physiology, 17, 1745959. 10.3389/fphys.2026.1745959 41983005 PMC13070835

[phy271054-bib-0016] Cirino, C. , Breda, F. L. , Polisel, E. E. C. , Lourenço, T. F. , Papoti, M. , Gobatto, C. A. , & Manchado‐Gobatto, F. B. (2025). Muscle oxygenation threshold in more and less active muscles and 3,000‐m running pace. International Journal of Sports Medicine, 46(12), 927–936. 10.1055/a-2611-3388 40570882

[phy271054-bib-0018] Di Prampero, P. E. (1981). Energetics of muscular exercise. In Reviews of Physiology, Biochemistry and Pharmacology (Vol. 89, pp. 143–222). Springer. 10.1007/BFb0035266 7015457

[phy271054-bib-0019] Engel, P. C. , & Jones, J. B. (1978). Causes and elimination of erratic blanks in enzymatic metabolite assays involving the use of NAD+ in alkaline hydrazine buffers: Improved conditions for the assay of l‐glutamate, l‐lactate, and other metabolites. Analytical Biochemistry, 88(2), 475–484. 10.1016/0003-2697(78)90447-5 29519

[phy271054-bib-0021] Faulkner, J. A. (1966). Physiology of swimming. Research Quarterly of the American Association for Health, Physical Education, and Recreation, 37(1), 41–54. 10.1080/10671188.1966.10614734 5217133

[phy271054-bib-0022] Feldmann, A. , Quaresima, V. , Perrey, S. , & Ferrari, M. (2026). Distinguishing muscle oxygen saturation from tissue oxygen saturation: Accuracy, limitations, and practical utility in muscle oximetry. Sports Medicine ‐ Open, 12(1), 61. 10.1186/s40798-026-01033-w 42234358 PMC13234098

[phy271054-bib-0100] Ferrari, M. , Muthalib, M. , & Quaresima, V. (2011). The use of near‐infrared spectroscopy in understanding skeletal muscle physiology: recent developments. Philosophical Transactions. Series A, Mathematical, Physical, and Engineering Sciences, 369(1955), 4577–4590. 10.1098/rsta.2011.0230 22006907

[phy271054-bib-0023] Foster, C. , Rodriguez‐Marroyo, J. A. , & Koning, J. J. (2017). Monitoring training loads: The past, the present, and the future. International Journal of Sports Physiology and Performance, 12(2), S22–S28. 10.1123/ijspp.2016-0388 28253038

[phy271054-bib-0024] Girard, O. , Mendez‐Villanueva, A. , & Bishop, D. (2011). Repeated‐sprint ability ‐ part I: Factors contributing to fatigue. Sports Medicine (Auckland, N.Z.), 41(8), 673–694. 10.2165/11590550-000000000-00000 21780851

[phy271054-bib-0025] Gladden, L. B. (2004). Lactate metabolism: A new paradigm for the third millennium. The Journal of Physiology, 558(1), 5–30. 10.1113/jphysiol.2003.058701 15131240 PMC1664920

[phy271054-bib-0026] Glaister, M. (2005). Multiple Sprint work. Sports Medicine, 35(9), 757–777. 10.2165/00007256-200535090-00003 16138786

[phy271054-bib-0027] Guidetti, L. , Rivellini, G. , & Figura, F. (1996). EMG patterns during running: Intra‐ and inter‐individual variability. Journal of Electromyography and Kinesiology: Official Journal of the International Society of Electrophysiological Kinesiology, 6(1), 37–48. 10.1016/1050-6411(95)00015-1 20719661

[phy271054-bib-0028] Halliwill, J. R. , Buck, T. M. , Lacewell, A. N. , & Romero, S. A. (2013). Postexercise hypotension and sustained postexercise vasodilatation: What happens after we exercise? Experimental Physiology, 98(1), 7–18. 10.1113/expphysiol.2011.058065 22872658

[phy271054-bib-0029] Herzog, S. , Philippi, M. , Feldmann, A. , Asian, J. , Filter, A. , Requena, B. , Batterson, P. M. , & Bogdanis, G. C. (2026). Muscle oxygen saturation as a marker of internal load in elite soccer: A case series. Journal of Strength and Conditioning Research. Publish Ahead of Print. 10.1519/JSC.0000000000005483 42336473

[phy271054-bib-0031] Impellizzeri, F. , Impellizzeri, F. M. , Marcora, S. M. , & Coutts, A. J. (2019). Internal and external training load: 15 years on. International Journal of Sports Physiology and Performance, 14(2), 270–273. 10.1123/ijspp.2018-0935 30614348

[phy271054-bib-0032] Jones, B. , Hesford, C. M. , & Cooper, C. E. (2013). The use of portable NIRS to measure muscle oxygenation and haemodynamics during a repeated sprint running test. Advances in Experimental Medicine and Biology, 789, 185–191. 10.1007/978-1-4614-7411-1_26 23852494

[phy271054-bib-0033] Joyner, M. J. , & Casey, D. P. (2015). Regulation of increased blood flow (hyperemia) to muscles during exercise: A hierarchy of competing physiological needs. Physiological Reviews, 95(2), 549–601. 10.1152/physrev.00035.2013 25834232 PMC4551211

[phy271054-bib-0034] Kitada, T. , Machida, S. , & Naito, H. (2015). Influence of muscle fibre composition on muscle oxygenation during maximal running. BMJ Open Sport & Exercise Medicine, 1(1), e000062. 10.1136/bmjsem-2015-000062 PMC5117045 27900139

[phy271054-bib-0036] Manchado‐Gobatto, F. B. , Marostegan, A. B. , Rasteiro, F. M. , Cirino, C. , Cruz, J. P. , Moreno, M. A. , & Gobatto, C. A. (2020). New insights into mechanical, metabolic and muscle oxygenation signals during and after high‐intensity tethered running. Scientific Reports, 10(1), 6336. 10.1038/s41598-020-63297-w 32286408 PMC7156678

[phy271054-bib-0038] McCully, K. K. , & Hamaoka, T. (2000). Near‐infrared spectroscopy: What can it tell us about oxygen saturation in skeletal muscle. Exercise and Sport Sciences Reviews, 28(3), 123–127. 10916704

[phy271054-bib-0040] McGawley, K. , & Bishop, D. J. (2015). Oxygen uptake during repeated‐sprint exercise. Journal of Science and Medicine in Sport, 18(2), 214–218. 10.1016/j.jsams.2014.02.002 24602687

[phy271054-bib-0041] McKee, J. R. , Girard, O. , Peiffer, J. J. , Dempsey, A. R. , Smedley, K. , & Scott, B. R. (2024). Continuous blood flow restriction during repeated‐sprint exercise increases peripheral but not systemic physiological and perceptual demands. European Journal of Sport Science, 24(6), 703–712. 10.1002/ejsc.12106 38874946 PMC11235999

[phy271054-bib-0042] Michailidis, Y. , Chatzimagioglou, A. , Mikikis, D. , Ispirlidis, I. , & Metaxas, T. (2020). Maximal oxygen consumption and oxygen muscle saturation recovery following repeated anaerobic sprint test in youth soccer players. Journal of Sports Medicine and Physical Fitness, 60(3), 355–360. 10.23736/S0022-4707.19.10162-4 31684709

[phy271054-bib-0043] Milioni, F. , Zagatto, A. M. , Barbieri, R. A. , Andrade, V. L. , Dos Santos, J. W. , Gobatto, C. A. , Da Silva, A. S. R. , Santiago, P. R. P. , & Papoti, M. (2017). Energy systems contribution in the running‐based anaerobic Sprint test. International Journal of Sports Medicine, 38(3), 226–232. 10.1055/S-0042-117722 28192833

[phy271054-bib-0045] Ogata, H. , Arimitsu, T. , Matsuura, R. , Yunoki, T. , Horiuchi, M. , & Yano, T. (2007). Relationship between oxygenation in inactive biceps brachii muscle and hyperventilation during leg cycling. Physiological Research, 56, 57–65. 16497096 10.33549/physiolres.930888

[phy271054-bib-0046] Orcioli‐Silva, D. , Beretta, V. S. , Santos, P. C. R. , Rasteiro, F. M. , Marostegan, A. B. , Vitório, R. , Gobatto, C. A. , & Manchado‐Gobatto, F. B. (2024). Cerebral and muscle tissue oxygenation during exercise in healthy adults: A systematic review. Journal of Sport and Health Science, 13(4), 459–471. 10.1016/j.jshs.2024.03.003 38462172 PMC11184313

[phy271054-bib-0047] Osawa, T. , Shiose, K. , & Takahashi, H. (2017). Delayed onset of reoxygenation in inactive muscles after high‐intensity exercise. In Oxygen Transport to Tissue XXXIX (Vol. 977, pp. 255–260). Springer. 10.1007/978-3-319-55231-6_35 28685454

[phy271054-bib-0048] Pereira, V. , Pereira, V. H. , Gobatto, C. A. , Lewis, T. G. , Ribeiro, L. F. P. , Beck, W. R. , Reis, I. G. M. , Sousa, F. A. B. , & Manchado‐Gobatto, F. B. (2018). Computational and complex network modeling for analysis of sprinter athletes' performance in track field tests. Frontiers in Physiology, 9, 843. 10.3389/fphys.2018.00843 30034346 PMC6043640

[phy271054-bib-0049] Perrey, S. , Quaresima, V. , & Ferrari, M. (2024). Muscle oximetry in sports science: An updated systematic review. Sports Medicine (Auckland, N.Z.), 54(4), 975–996. 10.1007/s40279-023-01987-x 38345731 PMC11052892

[phy271054-bib-0050] Rissanen, A.‐P. E. , Tikkanen, H. O. , Koponen, A. S. , Aho, J. M. , Hägglund, H. , Lindholm, H. , & Peltonen, J. E. (2012). Alveolar gas exchange and tissue oxygenation during incremental treadmill exercise, and their associations with blood O_2_ carrying capacity. Frontiers in Physiology, 3, 265. 10.3389/fphys.2012.00265 22934021 PMC3429041

[phy271054-bib-0052] Sendra‐Pérez, C. , Encarnacion‐Martinez, A. , Salvador‐Palmer, R. , Murias, J. M. , & Priego‐Quesada, J. I. (2025). Profiles of muscle‐specific oxygenation responses and thresholds during graded cycling incremental test. European Journal of Applied Physiology, 125(1), 237–245. 10.1007/s00421-024-05593-1 39259396 PMC11752943

[phy271054-bib-0053] Simpson, C. , Simpson, C. W. C. , Walter, J. , Gieseg, S. P. , Lackner, S. , Holasek, S. , & Hamlin, M. J. (2025). Central and peripheral nervous system activity and muscle oxygenation in athletes during repeated‐sprint exercise in normoxia and normobaric hypoxia. Journal of Sports Sciences, 43(19), 2204–2216. 10.1080/02640414.2025.2461947 39912708

[phy271054-bib-0054] Smith, K. J. , & Billaut, F. (2010). Influence of cerebral and muscle oxygenation on repeated‐sprint ability. European Journal of Applied Physiology, 109(5), 989–999. 10.1007/s00421-010-1444-4 20354718

[phy271054-bib-0055] Sousa, F. , Dos Reis, I. , Ribeiro, L. , Martins, L. , & Gobatto, C. (2015). Specific measurement of tethered running kinetics and its relationship to repeated Sprint ability. Journal of Human Kinetics, 49, 245–256. 10.1515/hukin-2015-0127 26839625 PMC4723174

[phy271054-bib-0057] Spencer, M. , Bishop, D. , Dawson, B. , & Goodman, C. (2005). Physiological and metabolic responses of repeated‐Sprint activities. Sports Medicine, 35(12), 1025–1044. 10.2165/00007256-200535120-00003 16336007

[phy271054-bib-0058] Tereso, D. , Gamonales, J. M. , Hernández‐Beltrán, V. , & Paulo, R. (2026). Assessment of internal load and external load in senior football players: Differences between competitive levels. Journal of Functional Morphology and Kinesiology, 11(2), 242. 10.3390/jfmk11020242 42346771 PMC13300932

[phy271054-bib-0059] Thurlow, F. , Huynh, M. , Townshend, A. , SJ, M. L. , James, L. P. , Taylor, J. M. , Weston, M. , & Weakley, J. (2024). The effects of repeated‐Sprint training on physical fitness and physiological adaptation in athletes: A systematic review and meta‐analysis. Sports Medicine (Auckland, N.Z.), 54(4), 953–974. 10.1007/s40279-023-01959-1 38041768

[phy271054-bib-0061] Turner, L. A. , Tecklenburg‐Lund, S. , Chapman, R. F. , Stager, J. M. , Duke, J. W. , & Mickleborough, T. D. (2013). Inspiratory loading and limb locomotor and respiratory muscle deoxygenation during cycling exercise. Respiratory Physiology & Neurobiology, 185(3), 506–514. 10.1016/j.resp.2012.11.018 23228896

[phy271054-bib-0062] Usher, A. , Babraj, J. , & Younger, A. (2025). Muscle oxygenation response during duplicate sprints in professional football players: An original investigation. Muscles, 4(4), 54. 10.3390/muscles4040054 41283442 PMC12641757

[phy271054-bib-0063] Vasquez‐Bonilla, A. A. , Camacho‐Cardeñosa, A. , Timón, R. , Martínez‐Guardado, I. , Camacho‐Cardeñosa, M. , & Olcina, G. (2021). Muscle oxygen desaturation and re‐saturation capacity limits in repeated Sprint ability performance in women soccer players: A new physiological interpretation. International Journal of Environmental Research and Public Health, 18(7), 3484. 10.3390/ijerph18073484 33801649 PMC8037739

[phy271054-bib-0064] Willis, S. J. , Borrani, F. , & Millet, G. P. (2019). Leg‐ vs arm‐cycling repeated sprints with blood flow restriction and systemic hypoxia. European Journal of Applied Physiology, 119(8), 1819–1828. 10.1007/s00421-019-04171-0 31187281

[phy271054-bib-0065] Wolf, M. , Ferrari, M. , & Quaresima, V. (2007). Progress of near‐infrared spectroscopy and topography for brain and muscle clinical applications. Journal of Biomedical Optics, 12(6), 062104. 10.1117/1.2804899 18163807

[phy271054-bib-0066] Woorons, X. , Dupuy, O. , Mucci, P. , Millet, G. P. , & Pichon, A. (2019). Cerebral and muscle oxygenation during repeated shuttle run sprints with hypoventilation. International Journal of Sports Medicine, 40(6), 376–384. 10.1055/a-0836-9011 30900226

[phy271054-bib-0067] Yogev, A. , Arnold, J. , Clarke, D. , Guenette, J. A. , Sporer, B. C. , & Koehle, M. S. (2022). Comparing the respiratory compensation point with muscle oxygen saturation in locomotor and non‐locomotor muscles using wearable NIRS spectroscopy during whole‐body exercise. Frontiers in Physiology, 13, 818733. 10.3389/fphys.2022.818733 35431982 PMC9007235

[phy271054-bib-0068] Yogev, A. , Arnold, J. , Nelson, H. , Clarke, D. C. , Guenette, J. A. , Sporer, B. C. , & Koehle, M. S. (2023). The effect of severe intensity bouts on muscle oxygen saturation responses in trained cyclists. Frontiers in Sports and Active Living, 5, 1086227. 10.3389/fspor.2023.1086227 36909360 PMC9995910

[phy271054-bib-0069] Zagatto, A. M. , Beck, W. R. , & Gobatto, C. A. (2009). Validity of the running anaerobic Sprint test for assessing anaerobic power and predicting short‐distance performances. Journal of Strength and Conditioning Research, 23(6), 1820–1827. 10.1519/JSC.0b013e3181b3df32 19675478

[phy271054-bib-0070] Zhang, Z. , Wang, B. , Gong, H. , Xu, G. , Nioka, S. , & Chance, B. (2010). Comparisons of muscle oxygenation changes between arm and leg muscles during incremental rowing exercise with near‐infrared spectroscopy. Journal of Biomedical Optics, 15(1), 017007. 10.1117/1.3309741 20210481

